# Gender-differences in antithrombotic therapy across the spectrum of ischemic heart disease: Time to tackle the Yentl syndrome?

**DOI:** 10.3389/fcvm.2022.1009475

**Published:** 2022-10-31

**Authors:** Renzo Laborante, Josip Andjelo Borovac, Mattia Galli, Daniele Rodolico, Giuseppe Ciliberti, Attilio Restivo, Luigi Cappannoli, Alessandra Arcudi, Rocco Vergallo, Andrea Zito, Giuseppe Princi, Antonio Maria Leone, Cristina Aurigemma, Enrico Romagnoli, Rocco Antonio Montone, Francesco Burzotta, Carlo Trani, Domenico D’Amario

**Affiliations:** ^1^Department of Cardiovascular and Pulmonary Sciences, Catholic University of the Sacred Heart, Rome, Italy; ^2^Department of Pathophysiology, University of Split School of Medicine, Split, Croatia; ^3^Maria Cecilia Hospital, GVM Care & Research, Cotignola, Italy; ^4^Department of Cardiovascular and Thoracic Sciences, Fondazione Policlinico Universitario Agostino Gemelli IRCCS, Rome, Italy

**Keywords:** antithrombotic therapy, antiplatelet therapy, anticoagulant therapy, ischemic heart disease, gender differences

## Abstract

The incidence and clinical presentation of ischemic heart disease (IHD), as well as thrombotic and bleeding risks, appear to differ between genders. Compared with men, women feature an increased thrombotic risk, probably related to an increased platelet reactivity, higher level of coagulation factors, and sex-associated unique cardiovascular risk factors, such as pregnancy-related (i.e., pre-eclampsia and gestational diabetes), gynecological disorders (i.e., polycystic ovary syndrome, early menopause) and autoimmune or systemic inflammatory diseases. At the same time, women are also at increased risk of bleeding, due to inappropriate dosing of antithrombotic agents, smaller blood vessels, lower body weight and comorbidities, such as diabetes and chronic kidney disease. Pharmacological strategies focused on the personalization of antithrombotic treatment may, therefore, be particularly appealing in women in light of their higher bleeding and ischemic risks. Paradoxically, although women represent a large proportion of cardiovascular patients in our practice, adequate high-quality clinical trial data on women remain scarce and inadequate to guide decision-making processes. As a result, IHD in women tends to be understudied, underdiagnosed and undertreated, a phenomenon known as a *“Yentl syndrome.”* It is, therefore, compelling for the scientific community to embark on dedicated clinical trials to address underrepresentation of women and to acquire evidence-based knowledge in the personalization of antithrombotic therapy in women.

## Introduction

In a 1983 movie, a young woman, named Yentl, attempted to live as a man to pursue the education she desired, blurring lines between traditional gender roles and deeply rooted social boundaries. In 1991, on the basis of this plot, Dr. Bernadine Healy coined the expression “Yentl syndrome” to epitomize the phenomenon in which women affected by ischemic heart disease (IHD) are less likely than men to receive recommended diagnostic tests, pharmacotherapy and invasive procedures, thereby showing a higher incidence of adverse outcomes (1). In the same year, as director of the National Institutes of Health, she launched the Women’s Health Initiative (WHI), consisting of an observational study and three clinical trials to address risk factors for cardiovascular disease, cancer and osteoporosis in postmenopausal women. The program is still ongoing and is expected to end in 2026, with over 160,000 women enrolled at present. Nevertheless, cardiovascular disease continues to be the leading cause of death among women, despite a considerable decline in cardiovascular deaths over several decades. In the last 30 years, cardiovascular research progressed significantly in order to achieve a personalized approach to care, including risk prediction models, preventive measures, and targeted therapeutic pathways. Antithrombotic therapy in patients undergoing percutaneous coronary intervention (PCI) has been deeply involved in this process. The propensity to ischemic recurrences after PCI and the understanding of prognostic implications associated with bleeding have prompted a substantial evolution in antithrombotic treatment regimens on the basis of a more accurate stratification of patients according to their ischemic and bleeding risks (2).

In this narrative review, the authors aim to explore the advancements and the limits of antithrombotic treatment in women, in the light of differences in epidemiology, clinical presentation, pathophysiology, bleeding, and ischemic risks among genders.

### Epidemiology of ischemic heart disease in women

Ischemic heart disease represents the principal cause of death in women globally, accounting for 35% of total deaths (3, 4). Women suffer from IHD approximately 5–10 years after men and have a 20% higher adjusted mortality risk in short term after successful PCI compared with men (5–7). Women with acute coronary syndromes (ACS) are more likely to present with non-ST-elevation acute myocardial infarction (NSTEMI), higher comorbidity burden at baseline and have less severe coronary atherosclerosis (8–12). Furthermore, women with ACS seek medical attention significantly later than men, thus also having prolonged door-to-balloon times (13, 14). The INTERHEART study revealed the importance of psychosocial risk factors, including depression, perceived stress at home or work, lower socioeconomic status, post-traumatic stress disorder and anxiety disorders, in the onset and clinical course of IHD (5, 15). They play a more significant role in women, due to a higher prevalence in this subset of patients. Notably, the impact of these risk factors on IHD are both direct, related to their pathophysiological consequences of the neuroendocrine and cardiovascular systems, and indirect, representing relevant predictors of non-adherence to medical treatment and unhealthy behaviors such as smoking and sedentary lifestyle (16–19). Furthermore, selective serotonin reuptake inhibitors (SSRIs), used as first-line drugs for many of the above conditions, have been demonstrated to impair hemostatic function through various mechanisms (i.e., blockade of intra-platelet calcium mobilization, depletion of intracellular serotonin and reduced secretion of platelet factors in response to chemical stimuli) and to increase the risk of bleeding (20). Conversely, certain SSRIs (i.e., fluoxetine and fluvoxamine) are potent inhibitors of CYP2C19, responsible for converting clopidogrel in its active form. In a large population-based cohort study of CYP2C19-inhibiting SSRI users (*n* = 9284) vs. non-CYP2C19-inhibiting SSRI users (*n* = 45,073), an increased risk of ischemic events was found in patients taking CYP2C19-inhibiting SSRIs (21).

Although classic type 1 acute myocardial infarction (AMI) occurs three times more commonly in men than in women, myocardial infarction in the absence of obstructive coronary arteries (MINOCA) is more common in women, being present in 10.5% of ACS presentations *vs.* 3.4% in men (22, 23). In women with MINOCA, mortality risk is significantly associated with the number of accompanying risk factors, ranging between 10% with ≤ 1 cardiovascular risk factor and 25% with > 3 risk factors (24).

Spontaneous coronary artery dissection (SCAD) is a rare cause of ACS, but 90% of the cases are reported in women and it accounts for 10–20% of AMI in women younger than 50 years of age (25, 26). Among the causes of MINOCA, vasospastic angina and microvascular angina play an important role. Whilst rest angina due to epicardial coronary arteries vasospasm is more common in men, the prevalence of coronary microvascular dysfunction among patients with chest pain and non-obstructive coronary artery disease is higher in women compared to men (27).

The prevalence of stress-induced cardiomyopathy, also known as Takotsubo syndrome (TS) has been reported to be approximately 2% of all patients presenting with clinical manifestation of ACS (28). Importantly, out of all TS cases, 90% of patients are post-menopausal women and it is estimated that this entity is present in 5–6% of all female patients presenting with suspected ST-elevation myocardial infarction (STEMI) (28).

In conclusion, although IHD has long been considered a disease affecting predominantly male patients, it constitutes also a considerable part among diseases affecting women. However, there are important differences in terms of clinical subtypes among men and women with ACS. Considering the high prevalence of MINOCA in women with ACS, a strategy of multimodality imaging assessment should be always pursued, using both invasive (i.e., provocative spasm test and intracoronary imaging -IVUS and OCT-) and non-invasive tests (i.e., echocardiogram and cardiac magnetic resonance) in order to identify the specific etiology and provide the right treatment option.

### Clinical presentation of ischemic heart disease in women

The presence of chest pain/discomfort is the hallmark symptom of IHD. A comprehensive analysis from the National Registry of Myocardial Infarction (NRMI), reporting hospital data on 1,143,513 registry patients admitted with confirmed AMI (481,581 women and 661,932 men), have demonstrated that women were more likely than men to present without chest pain (42.0% *vs.* 30.7% in men, respectively), with a larger sex difference in younger patients (29). Women, especially under the age of 65, show more frequently a wide spectrum of atypical symptoms, including weakness, fatigue, nausea, dyspnea, as well as unconventional event triggers (i.e., mental or emotional stress instead of physical exertion) and locations of chest-related symptoms, such as in the neck, jaw, and in the back (30). The reasons for sex-based differences in IHD symptom presentation are largely unknown. A possible explanation could be that younger women who experience AMI may have significantly less narrowing of the coronary arteries than older women or men due to a hypercoagulable state, inflammation, coronary spasm or plaque erosion instead of rupture (31). Furthermore, women exhibit differences in the neural receptors and pathways involved in nociception (32).

Such characteristics demonstrate that women who suffer from IHD may represent a heterogeneous patient group compared to men, requiring both an adaptation of diagnostic criteria and tailored medical anti-ischemic therapy due to a different underlying pathophysiology of coronary disease.

### Sex differences in platelet function

Platelets are blood cells with several important biological functions as they regulate the integrity of the vascular wall, play a key role in primary hemostasis, and modulate thrombotic and inflammatory responses at the blood-vascular interface (28). Sexual dimorphism and age differences in human platelet aggregation dynamics have been known for several decades (33). Such findings may be of clinical relevance since antiplatelet therapy is the fundamental constituent in the treatment of IHD and might require sex-specific tailoring (33).

In a work by Becker et al., women presented a higher platelet reactivity to arachidonic acid and adenosine diphosphate (ADP) at baseline and after treatment with low-dose aspirin, although they experienced the same or greater decreases in platelet reactivity after treatment (34). Similarly, Gremmel et al. found that women were associated with a more pronounced formation of leukocyte-platelet aggregates and increased protease-activated receptor mediated platelet reactivity after PCI (35). The higher platelet reactivity in women was proposed also in patients undergoing double anti-platelet therapy (DAPT) with aspirin and clopidogrel, using thrombin receptor-activating peptide as a stimulator (36). Furthermore, in a cohort of 760 patients undergoing cardiac surgery, clopidogrel-treated women had higher platelet reactivity (HRP) to ADP (37). Similarly, recent data from Myocardial Ischemia Detection By Circulating Biomarkers (MYOMARKER) study showed attenuated flow-citometry-based platelet reactivity to P_2_Y_12_ inhibitor (mainly clopidogrel) among female outpatients with suspected myocardial ischemia when compared to men (38). Moreover, a recent analysis of 177 participants on clopidogrel after ACS, showed that the risk of an atherothrombotic event was greater in female carriers loss-of-function allele, compared to men carriers of the same allele, suggesting a possible interaction between sex and genes for clopidogrel (39). The potential increased platelet reactivity in women may be due to multiple causes, such as a higher platelet count and higher number of surface receptors in females in general which points to a greater agonist-induced platelet activation and aggregation (30). However, other studies opposed the previous ones, suggesting an equal platelet response to aspirin and P2Y12 inhibitors (40, 41–43).

However, the clinical implications of these findings remain unclear. Although the occurrence of major adverse cardiovascular and cerebrovascular events (MACCE) was significantly correlated to HPR, a recent meta-analysis, evaluating cardiovascular efficacy of clopidogrel, opposes the above-mentioned results and suggests no significant difference in treatment efficacy between men and women (44, 45). Similarly, two meta-analyses found no evident differences in clinical outcomes between sexes in patient treated with cardioaspirin (46, 47).

**FIGURE 1:**
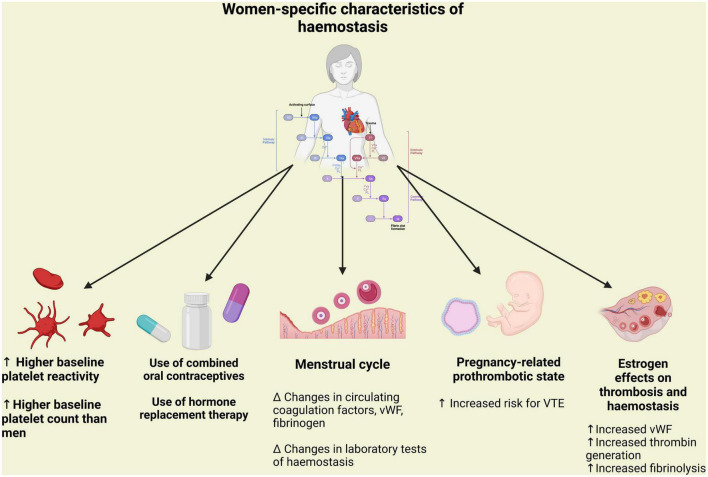
Peculiarity of hemostasis in women in terms of platelet aggregation and coagulation associated with pregnancy and hormonal status. vWF, Von Willebrand factor; VTE, venous thromboembolism.

Taken together, women seem to have higher platelet reactivity than men at baseline, whereas conflicting data have been reported regarding platelet response to aspirin and P2Y12 inhibitors (Figure 1). Future studies are needed to determine if the possible sex difference in platelet reactivity could be addressed by the use of newer or different dosages of antiplatelet agents and if this will portend any impact on relevant clinical endpoints.

### Sex differences in coagulation

The coagulation cascade of secondary hemostasis is constituted by a series of reactions catalyzed by different enzymes, known as coagulation factors, ultimately resulting in cross-linked fibrin (48). This process is considerably influenced by fluctuations in hormone status associated with the menstrual cycle, pregnancy, menopause, hormone-based contraceptives and hormone replacement therapy (HRT) preparations (49). A cyclic variations of von Willebrand factor (VWF), fibrinogen, and activated factor VII have been reported during the normal menstrual cycle. Moreover, pregnancy and the oral administration of synthetic estrogens are associated with a progressive increase in the levels of procoagulant factors, VWF and fibrinogen and to a reduction in the activity of some coagulation regulatory proteins (tissue factor pathway inhibitor, protein S, protein C and antithrombin), leading to a hypercoagulable state (Figure 1) (49). These hormonal influences increase significantly the risk of venous thromboembolism, whereas their association with a higher risk of arterial thrombosis is still a matter of debate (31). Caution should be warranted when interpreting data on sex differences in platelet function and coagulation, given the heterogeneity of *in vitro*, *ex vivo* and *in vivo* studies, the multiple clinical scenarios (i.e., pre-/post-menopausal states or pregnancy) and the different dosages, routes of administration and combinations of hormone-based therapies.

### Thrombotic risk in women within the spectrum of ischemic heart disease

Recently, the applicability of traditional risk factors in women (i.e., diabetes, smoking) has been questioned, as the majority of studies are predominantly conducted in the male population. Sex differences in the relative excess of cardiovascular risk associated with diabetes mellitus (DM) have been reported in several studies and have been confirmed by a recent meta-analysis of individual data from 980,793 adults; this analysis showed that women with DM exhibited a three-fold increased risk of cardiovascular mortality, whereas DM only doubles cardiovascular mortality risk in men (50). To date, the reason of this relative excess risk in women associated with the presence of DM is not elucidated.

**FIGURE 2:**
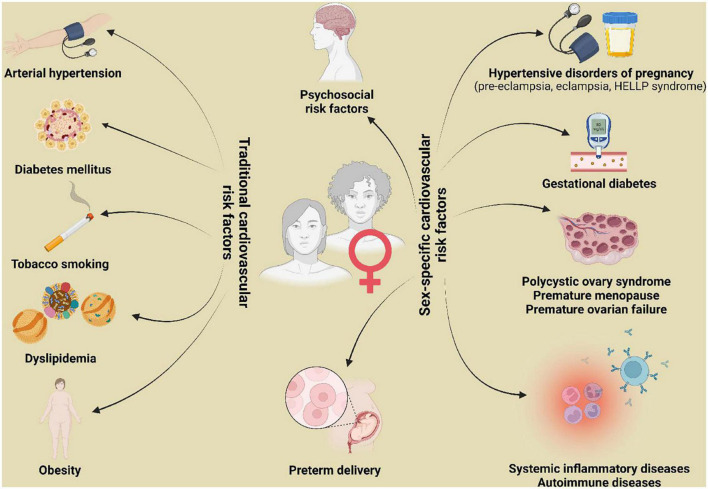
Traditional and sex-specific cardiovascular risk factors. HELLP, hemolysis, elevated liver enzymes and low platelets.

Similarly, the impact of smoking on the development of IHD seems to be greater in women than in men (51). A recent meta-analysis including 2.4 million individuals reported that female smokers have a 25% greater risk of IHD compared with male smokers (52). Furthermore, obesity has a greater prognostic impact on women compared to men. In fact, the Framingham Heart Study showed that obesity increased the relative risk of IHD by 64% in women, as opposed to 46% in men (53). Data from 15,624 Norvegian individuals revealed that a similar increase in male or female body-mass index (BMI) was associated with a greater increase in systolic blood pressure in women than in men (54). However, BMI cannot be used as a comparable measure of fat tissue distribution between sexes, because it cannot discriminate between fat and fat-free mass. In fact, women result to have significantly greater amounts of total body fat than men with an equivalent BMI (55). Indeed, the pattern of lipid accumulation differs in women and men: women more often develop peripheral adiposity, with gluteal fat accumulation, whereas men are more prone to central or android obesity. However, after menopause, body fat distribution shifts to a more male pattern. Central fat, unlike peripheral adiposity, releases inflammatory mediators, which affect glucose and fat metabolisms and contribute to the development of metabolic syndrome (56). Nevertheless, BMI does not reflect fat distribution, as it is an exclusively quantitative parameter. In summary, BMI alone is not sufficient to properly assess the cardiometabolic risk associated with increased adiposity in women and other strategies, such as waist circumference measurement and bioimpedance analysis, should be implemented (57). Apart from traditional cardiovascular risk factors, there are a number of clinical conditions unique to women that have been identified to be associated with increased thrombotic risk. These include pregnancy disorders, such as pre-eclampsia, eclampsia, and gestational diabetes, gynecological disorders (i.e., polycystic ovary syndrome, early menopause), autoimmune and/or systemic inflammatory disease, known to disproportionally affect women compared to men (49) (Figure 2). As confirmation of the prognostic impact of non-traditional risk factors, nearly 20% of all coronary events occur in the absence of any traditional risk factors in women (51). Unfortunately, acquired awareness in thrombotic risk has not yet translated into changes in standard clinical care.

Double anti-platelet therapy score is the only tool endorsed by European and North-American guidelines to assess specifically thrombotic risk after PCI, identifying patients expected to derive benefit from continuing P_2_Y_12_ inhibitors beyond 1 year after PCI (58, 59). It was developed from the DAPT trial and validated in the PROTECT trial, but the proportion of women participating in each trial was subpar – 27 and 24%, respectively (60, 61). Even more, recent studies have shown women are less often prescribed antiplatelet therapy for secondary prevention, compared to men (51). Further outreach and awareness raising are necessary to ensure that gender with associated unique cardiovascular risk factors are included as important modifiers in thrombosis risk stratification scores, in order to guide clinicians in tailoring antithrombotic therapy after PCI, in terms of duration and intensity.

### Strategies aimed at reducing ischemic events

Multiple strategies focused on reducing the residual burden of ischemic events among patients at high ischemic risk, undergoing PCI, have been developed over the years (2). These include the use of newer P_2_Y_12_ inhibitors (i.e., prasugrel, ticagrelor, and cangrelor) instead of clopidogrel or the addition of GP IIb/IIIa inhibitors (GPI), prolonging DAPT duration and the addition of a novel oral anticoagulants (NOACs) to standard antiplatelet treatment regimens, a strategy also known as dual pathway inhibition (DPI) (2, 62–70). As mentioned above, women represent a category with a higher ischemic burden compared to men with similar cardiovascular risk factors. Therefore, women could potentially benefit from these strategies even more than men, although robust evidence is currently lacking due to low percentage of women enrolled in trials (Table 1). The CURE (Clopidogrel in Unstable Angina to Prevent Recurrent Ischemic Events) trial, evaluating the addition of clopidogrel to aspirin in 12,562 patients with NSTEMI, showed women presented a smaller relative risk reduction (12% *vs.* 25%) in the composite endpoint of cardiovascular death, non-fatal AMI, or stroke compared with men at 1-year follow-up (71). Similar results were found in the subgroup of patients undergoing PCI (71). A subsequent meta-analysis of all blinded randomized clinical trials (RCTs) comparing clopidogrel and placebo and involving a total of 79,613 patients, confirmed the reduced efficacy in women compared to men: clopidogrel reduced only the risk of AMI and not that for stroke or all-cause mortality in women, whereas it reduced significantly all three endpoints in men (72, 73). Concerning ticagrelor and prasugrel, the PLATO trial and the TRITON-TIMI 38 trial, respectively, showed a similar reductions in the primary endpoint both in women and men, although these studies were not powered to examine treatment interactions among subgroups (74, 75). Similarly, two meta-analysis of randomized trials about PCI with adjunctive use of irreversible GPI (i.e., abciximab) or reversible GPIs (i.e., tirofiban or eptifibatide), demonstrated a similar efficacy both in men and women with no sex difference in terms of major adverse outcomes (76, 77). Accordingly, in a prespecified subgroup analysis of the CHAMPION PHOENIX trial, cangrelor demonstrated a similar reduction in the odds of major adverse cardiovascular events (MACE) in both sex (78).

**TABLE 1:** List of major randomized controlled trials evaluating antiplatelet strategies focused on reducing ischemic events with sub-group analysis by sex.

Name of study, year of publication	Drugs compared	Total patients	Number of women%	Primary outcome (reached?)	Gender difference in primary outcome
**Clopidogrel**					
CURE, 2001	Clopidogrel + aspirin vs. aspirin	12,562	38	Death, non-fatal MI, stroke (yes)	Yes: lower primary outcomes among men taking clopidogrel
CREDO, 2002	Clopidogrel + aspirin vs. aspirin	2,116	28.6	Death, non-fatal MI, stroke (yes)	Yes: lower primary outcome among men taking clopidogrel
**Potent P2Y12 inhibitors**					
TRITON-TIMI 38, 2007	Prasugrel + aspirin vs. clopidogrel + aspirin	13,608	5.8	Death, MI, stroke (yes)	Yes: lower primary outcomes among men taking prasugrel
PLATO, 2009	Ticagrelor + aspirin vs. clopidogrel + aspirin	18,624	28.3	Death, MI, stroke (yes)	No
ALPHEUS, 2020	Ticagrelor + aspirin vs. clopidogrel + aspirin	1,910	21	PCI-related type 4 (a or b) MI or major myocardial injury (no)	No
**Prolonging DAPT duration**					
DAPT, 2014	12 months versus 30 months DAPT	9,960	25.4	ST, death, MI or stroke (yes)	Yes: lower primary outcome among men treated with 30 months DAPT
PEGASUS, 2015	Ticagrelor + aspirin vs. clopidogrel + aspirin	21,162	23.6	Death, MI, stroke (yes)	No
THEMIS, 2020	Ticagrelor + aspirin vs. placebo + aspirin among stable patients with DM	19,220	31.4	Cardiovascular death, MI or stroke (yes)	Yes: lower primary outcome among men treated with ticagrelor
**Guided escalation of P2Y12 inhibitors**					
GRAVITAS, 2011	High-dose clopidogrel (150 mg) versus standard dose clopidogrel (75 mg) among clopidogrel non- responders	2,214	35	Cardiac death, non-fatal MI, or ST (no)	No
ARCTIC, 2012	High-dose prasugrel versus standard dose clopidogrel (75 mg) among clopidogrel non- responders	2,440	19	All death, MI, ST, stroke and urgent revascularization (no)	No
PATH-PCI, 2019	Ticagrelor among clopidogrel non-responders versus standard therapy	2,285	17	Cardiac death, MI, stroke, ST, urgent revascularization and bleeding (BARC 2,3 or 5)	Yes: lower primary outcome among men treated with ticagrelor
TAILOR PCI, 2020	Ticagrelor among clopidogrel non-responders versus standard therapy	5,302	25	No	No

BARC, Bleeding Academic Research Consortium; MI, myocardial infarction; PCI, percutaneous coronary intervention; ST, stent thrombosis.

Another strategy to reduce ischemic recurrences in patients at high ischemic risk, particularly those with prior AMI, is represented by prolongation of DAPT duration beyond 1 year. Dual Antiplatelet Therapy (DAPT) study, enrolling 9,961 patients, demonstrated, for the first time, that 30-month DAPT (with either clopidogrel 75 mg or prasugrel 10 mg) significantly reduced the primary endpoint of MACE, compared to 12-month DAPT (60). In a sub-group analysis, women randomly assigned to prolonged DAPT had a similar treatment effect for reduction in ischemic risk compared with males (79). Finally, the last strategy focused on reducing ischemic events is represented by DPI. To date, the low-dose rivaroxaban is so far the only NOAC to have been successfully tested as part of a DPI strategy in a phase III trial in patients with ACS (80). In particular, for patients with a recent ACS, low-dose rivaroxaban on top of standard of care antiplatelet therapy, most commonly aspirin and clopidogrel, reduced the risk of MACE and this benefit was significantly consistent only in the female subgroup of patients (81). In aggregate, a clear trend to a higher incidence of ischemic complications has been consistently reported in women. Strategies focused on reducing ischemic events appear to be equally effective in men and women, although the majority of trials are underpowered to assess differences in sex-specific subgroup analysis. Indeed, the main drawback of such intensive antithrombotic therapies is an enhanced risk of bleeding. It is, therefore, compelling for the scientific community to embark on dedicated clinical trials that will be equally inclusive to women as they are to men.

### Bleeding risk in women within the spectrum of ischemic heart disease

Bleeding events have a significant downstream impact on mortality and morbidity outcomes among patients undergoing PCI (82). Sex-related differences have been observed also in terms of bleeding risk. Data from 24,045 patients with ACS from the GRACE registry showed that female sex was significantly associated with a higher risk of bleeding (adjusted odds ratio of 1.43), even after controlling for the influence of other variables, including age, antithrombotic therapies, performance of invasive procedure and clinical presentation (83). These findings have been recently confirmed by a recent analysis of 4 post-approval ACS registries showing that the prevalence of high bleeding risk (HBR) according to the Bleeding Academic Research Consortium (BARC) definition was higher in women compared to men, with a consequent higher rate of major bleeding at 4 years (84).

This phenomenon can have multiple explanations. First, women tend to be older and more likely to have comorbidities such as diabetes, cronic kidney disease and hypertension at the time of IHD – these are well-known risk factors for future hemorrhagic events (32). Second, women have a higher risk for the development of vascular complications following PCI, probably due to smaller blood vessels in women, as well as difference in vascular reactivity (85). Finally, women may have a tendency to receive inappropriate dosing of antithrombotic agents, because no difference in dose recommendation currently exist, although women have, at least in part, a lower body weight, an older age, and a higher rate of renal insufficiency, despite similar serum creatinine levels, compared to men (30).

In order to estimate bleeding risk in patients with IHD, European Society of Cardiology (ESC) and North-American guidelines recommend the use of several scores, such as the CRUSADE score, the ACUITY score, the PARIS score and the ARC-HBR criteria. Furthermore, PRECISE-DAPT score have been designed to guide and inform decision making for patients on DAPT following PCI, integrating both ischemic and bleeding risks (86). Surprisingly, female sex appears only in the CRUSADE and in the ACUITY scores among predictor variables, although it clearly represents a risk factor for bleeding after PCI.

In conclusion, in the last two decades, a remarkable amount of data has consistently demonstrated sex-related differences in bleeding risk after PCI. Nevertheless, it has not translated into the adoption of standardized different recommendations, according to patient’s sex. This uncertainty is reflected by international recommendations on duration of DAPT, with female sex being included among the bleeding risk factors in the North-American but not the European guidelines (58, 87).

### Strategies aimed at reducing bleeding events

Bleeding has been recognized as a prognostically unfavorable event to the same extent as having a new or recurrent ischemic or thrombotic complication (88). The risk of bleeding tends to be stable over time while ischemic risk decreases after 1–3 months post-PCI, with a variability according to the clinical presentation of the patients and the complexity of the procedure (89). Therefore, after 1–3 months post-PCI, a series of pharmacological strategies can be implemented in order to reduce bleeding, possibly yielding a more favorable balance between bleeding and ischemic risk.

These strategies might include shortening of DAPT duration, the use of P_2_Y_12_ monotherapy and de-escalation of P_2_Y_12_ inhibitors (2, 90). Although they may be particularly appealing in women in light of their higher bleeding risk, these strategies are not extensively investigated in this subset of patients (Table 2). In addition, there are other non-pharmacological bleeding avoidance strategies, such as vascular closure device application, the use of radial access or the combination of these (88). Of note, the use of radial access resulted in a decrease in the rate of bleeding events to a greater extent in women, compared to men (91).

**TABLE 2:** List of major randomized controlled trials evaluating antiplatelet strategies focused on reducing bleeding events with sub-group analysis by sex.

Name of study, year of publication	Drugs compared	Total patients	Number of women%	Primary outcome (reached?)	Gender difference in primary outcome
**Shortening DAPT**					
ISAR-SAFE	6 versus 12 months DAPT	2,015	19.4	All-cause death, MI, ST, stroke, and TIMI major bleeding (yes)	No
NIPPON, 2017	6 versus 18 months DAPT	3,773	21	All-cause death, MI, stroke and major bleeding (yes)	No
SMART DATE, 2018	6 versus 12 months DAPT	2,712	25	All-cause death, MI or stroke (yes)	No
One-month DAPT, 2021	1 versus 6–12 months DAPT in non-complex PCI	3,020	31	Cardiac death, non-fatal MI, target vessel revascularization, stroke or major bleeding (yes)	No
MASTER DAPT, 2021	1 versus 5 months DAPT in HBR patients	4,434	30.7	All-cause death, MI, stroke or major bleeding (yes)	No
**P2Y12 monotherapy**					
GLOBAL LEADERS, 2018	Ticagrelor plus aspirin for 1 month, followed by ticagrelor monotherapy versus aspirin plus clopidogrel or ticagrelor for 12 months, followed by aspirin monotherapy	15,968	23	All-cause death and MI (yes)	No
TWILIGHT, 2019	Ticagrelor monotherapy after 3 months DAPT versus DAPT in high-risk PCI	7,119	23.8	BARC (2, 3, or 5) bleeding and all-cause death, non-fatal MI or non-fatal stroke (yes)	No
SMART-CHOICE, 2019	P2Y12 monotherapy after 3 months DAPT versus standard DAPT	2,993	26	All-cause death, MI or stroke (yes)	No
STOPDAPT-2, 2019	Clopidogrel monotherapy after 1 month of DAPT versus standard DAPT	3,045	22	Cardiac death, MI, stroke, ST, bleeding (yes)	No
TICO, 2020	Ticagrelor monotherapy after 3 months of DAPT versus standard DAPT	3,056	20.5	Major bleeding, death, MI, ST, stroke, or target-vessel revascularization (yes)	Yes: lower primary outcomes among women taking ticagrelor monotherapy
STODAPT-2-ACS, 2021	Clopidogrel monotherapy after 1 month of DAPT versus standard DAPT among ACS	4,169	22	Cardiac death, MI, any stroke, definite ST or bleeding (no)	No
**Guided de-escalation**					
ANTARTIC, 2016	Guided de-escalation versus standard DAPT	877	39	Cardiac death, MI, stroke, ST, urgent revascularization and BARC (types 2, 3, or 5) bleeding (no)	No
TROPICAL-ACS, 2017	Guided de-escalation versus standard DAPT	2,610	21.5	Cardiac death, MI, stroke and BARC (types 2, 3, or 5) bleeding (yes)	No
POPular genetics, 2019	Guided de-escalation versus standard DAPT	2,488	25	All-cause death, MI, ST, stroke or major bleeding (yes)	No
**Unguided de-escalation**					
TOPIC, 2017	Clopidogrel-based DAPT versus standard DAPT	646	18	Cardiac death, urgent revascularization, stroke and BARC (2, 3, or 5) bleeding (no)	No
HOST-REDUCE-POLYTECH-ACS, 2020	Prasugrel 5 mg-based DAPT versus prasugrel 10 mg-based DAPT	3,429	10.7	All-cause death, non-fatal MI, ST, repeat revascularization, stroke and BARC (2, 3 or 5 bleeding) (yes)	No
TALOS-MI, 2021	Ticagrelor among clopidogrel non-responders versus standard therapy	2,697	16.5	Cardiac death, MI, stroke, ST, urgent revascularization and bleeding (BARC 2, 3, or 5)	Yes: lower primary outcome among men treated with ticagrelor

BARC, Bleeding Academic Research Consortium; MI, myocardial infarction; PCI, percutaneous coronary intervention; ST, stent thrombosis; TIMI, thrombolysis in myocardial infarction.

Shortening of the DAPT duration has been the most largely investigated strategy and traditionally consists of the withdrawal of the P_2_Y_12_ inhibitor at the time earlier than conventional (12 months post-ACS) (2, 92–96). Sawaya et al. (97) pooled individual patient data from six RCTs comparing short- (≤6 months) versus long-term (≥1 year) DAPT after PCI. They showed short-term DAPT is associated with similar rates of MACE but lower risk of bleeding when compared with prolonged DAPT, with no significant difference between sexes. Although the hazard ratio of any bleeding and major bleeding suggest benefit in the subgroup of women, the *p*-value did not reach statistical significance. This is likely due to the fact that women were largely underrepresented in the above RCTs (about 30% of the overall population) thus not allowing a formal statistical power to be reached (97). Another important finding of this patient-level meta-analysis is that patient factors (ACS and diabetes) and lesion complexity (number of lesions stented and number of stents used) predicts the occurrence of MACE in women, underlying the importance of an accurate baseline risk stratification (97).

In the past 5 years, early aspirin discontinuation in patients undergoing PCI has emerged as a potential strategy to reduce bleeding without any increase in thrombotic events. To date, six RCTs with 36,350 patients (23.3% patients were female) have compared DAPT versus P_2_Y_12_ inhibitor monotherapy after a short duration of DAPT in patients after PCI (2, 98–102). An individual patient data meta-analysis of these six RCTs has been performed and showed that the use of P_2_Y_12_ monotherapy was associated with a significant reduction in the rate of bleeding without any increase in the rate of ischemic events (103). Interestingly, they investigated the consistency of these findings according to sex. Concerning the bleeding events, the treatment effect was consistent both in male and female patients, with a statistically significant reduction in both groups (103). Surprisingly, they observed a treatment-by-subgroup interaction with sex suggesting that P_2_Y_12_ inhibitor monotherapy lowers the risk of the primary ischemic endpoint in women but not in men (103). Whether this depends on a different response to aspirin and/or P_2_Y_12_ inhibitor remains the matter of debate. Female patients represent a subgroup, therefore, this finding should be considered hypothesis-generating only, due to intrinsic methodological and statistical limitations of subgroup analyses.

De-escalation of P_2_Y_12_ inhibiting therapy consists in switching from more potent (i.e., prasugrel or ticagrelor) to less potent (i.e., clopidogrel) agents, in order to reduce bleeding without any trade-off in ischemic events (104). De-escalation can be un-guided or guided through the aid of platelet function or genetic tests (105–108). A guided de-escalation strategy has been investigated in three RCTs, using either platelet function testing (*n* = 2) or genetic testing (*n* = 1) (109–112). TROPICAL ACS trial showed that guided de-escalation was non-inferior for the primary composite endpoint of net adverse cardiovascular events (NACE) as compared to standard of care, with a trend, although not statistically significant, toward reduced bleeding at 12 months compared to the standard group. Furthermore, a prespecified analysis of the TROPICAL-ACS trial investigated the impact of sex on clinical outcomes and found no significant interaction of sex with combined endpoint, ischemic events and bleeding.

POPular GENETICS trial showed that genotype-guided strategy was non-inferior for NACEs and superior in terms of PLATO major or minor bleeding, as compared to standard of care at 12-month follow-up. However, the reduction of bleeding became statistically insignificant in the subgroup of female patients, due to a relatively small sample size, which resulted in a broad 95% confidence interval.

Finally, it is worth considering the possible impact of herbal therapies on hemostasis and, consequently, on bleeding events. Multiple surveys have shown that women (especially white, middle-aged women, with good sociocultural status) are likely to be users of unconventional therapies, among which herbs play a prominent role (113). One of the most used is Ginkgo biloba, a species of tree native to China, from which an extract is obtained. It is commonly used as an antioxidant, to treat claudication intermittens and vascular dementia, although there is no evidence for its beneficial effects. Since it antagonizes platelet-activating factor, it predisposes to bleeding, especially in patients on aspirin or warfarin (114).

In short, it has been well-established for decades that women are at greater risk of bleeding. However, although various pharmacological strategies have been developed to minimize this risk, none of these have been extensively tested in female population. Therefore, evidence on their safety and efficacy in this subset of patients is lacking.

## Specific clinical conditions

### Atrial fibrillation

The prevalence and incidence of atrial fibrillation (AF) has been increasing in both sex over time (115). The number of women and men with AF are similar, despite the higher risk of AF in men, due to women’s increased longevity (115). AF increases the risk of stroke fivefold, but this risk is not homogeneous, depending on the presence of specific stroke risk factors. Common stroke risk factors are summarized in the CHA_2_DS_2_-VASc score, among which female sex is included (115). Of note, female sex has to be considered a stroke risk modifier rather than a risk factor *per se* (115). In the absence of other risk factors, women have a stroke risk similar to men, whereas women with other risk factors have significantly higher stroke risk than men (116). Women affected by AF and concomitant IHD are on average older and with more comorbidities than their male counterparts (117). Nevertheless, although they are at greater risk for stroke than men, they are significantly less likely to receive oral anticoagulants at all levels of the CHA_2_DS_2_-VASc score, paradoxically (118). The efficacy and safety of NOACs have been broadly demonstrated in overall population, even within 5 days after cardioembolic stroke (119, 120).

Sex differences in the efficacy and safety of warfarin compared to NOACs have long been investigated. According to a meta-analysis of 26,260 patients, women with AF have a significantly greater residual risk of systemic thromboembolism (STE) when treated using warfarin, whereas women treated with NOACs are at equivalent residual risk of STE and less major bleeding risk compared with men (121). Therefore, NOACs should be the anticoagulants of choice even more than in men.

Since NOACs have a different pharmacological profile compared to vitamin K antagonists, they may differ from one another in their effects on women with AF. An indirect comparison of them was performed, using data from foundational anticoagulant trials such as ROCKET-AF, RE-LY, ENGAGE-AF-TIMI and ARISTOTLE in which warfarin was used as an indirect comparator. No significant difference was found for any NOAC in terms of safety and efficacy in women with AF (122). Thus, a recent consensus document of the European Heart Rhythm Association (EHRA) indicates that the choice of the type of NOAC in females should follow general principles set for the overall population (123).

Nevertheless, data from adequately powered RCTs are needed to reach high quality evidence in the use of NOACs in women with concomitant AF for the prevention of STE. In general, 10–15% of AF patients undergo PCI for IHD and guidelines recommended TAT (triple antithrombotic therapy) for a certain time period after PCI in AF patients (115). However, there is still uncertainty whether TAT or double antithrombotic therapy (DAT) should be the first line choice for the majority of patients after hospital discharge. Holm et al. conducted an analysis on 272 patients discharged with TAT registered in the SWEDEHEART registry and showed that women discontinued TAT prematurely due to bleeds to a very high extent compared to men (124). Despite this, the rate of coronary events did not differ between sexes, although the study was underpowered to assess a possible sex difference in association between TAT discontinuation and ischemic events (124). To date, there are no sex analyses derived from RCTs regarding DAT and TAT to guide in treatment strategies, since data from RCTs were not powered to assess MACE, nor even differences among sex-specific subgroups (125–127).

### Spontaneous coronary artery dissection

Spontaneous coronary artery dissection is the most common cause of pregnancy-associated AMI and represents 35% of ACS cases among women under the age of 50 (49). The gold standard for diagnosis of SCAD is coronary angiography (128). However, the use of intravascular imaging, such as optical coherence tomography (OCT) or intravascular ultrasound (IVUS), could be useful to differentiate SCAD from atherosclerotic plaque, when diagnostic uncertainty exists, or to guide coronary intervention, when clinically required (128).

There seems to be a general consensus indicating that the initial conservative medical management is appropriate in most SCAD cases, whereas interventional treatment (i.e., PCI or coronary artery bypass grafting, CABG) should be considered in selected cases such as SCAD complicated by refractory ongoing ischemia, hemodynamic instability or sustained ventricular tachyarrhythmias (128).

The antiplatelet regimen to be used in patients treated conservatively is still a matter of debate, since there are no RCTs comparing different pharmacological treatment strategies for SCAD. Whilst DAPT is the most commonly prescribed strategy in SCAD (usually with aspirin and clopidogrel rather than newer P_2_Y_12_ inhibitors), recent data from DISCO registry showed that DAPT was associated with a higher rate of MACE at 12 months of follow-up, driven by an early excess of non-fatal AMI or unplanned PCI (129). DAPT may cause enhancement of intramural bleeding, with subsequent propagation of the dissection and higher rate of adverse events. To support this hypothesis, Garcia-Guimaraes et al. reported that the presence of long intramural hematoma (>20 mm) is an independent predictor of in-hospital MACE in patients treated with DAPT (130). Therefore, DAPT may be actually harmful in conservatively managed SCAD patients, especially in those with contained IMH (i.e., type 2 SCAD). In case of SCAD occurring during pregnancy, particular attention should be paid to the choice of antithrombotic drugs, due to potential adverse effects to fetus (Table 3).

**TABLE 3:** Safety of anti-thrombotic drugs during pregnancy and breastfeeding.

Drugs	Risk category	Placenta permeable	Transfer to breast milk	Safety data
**Antiplatelet drugs**				
Abciximab	C	Unknown	Unknown	Inadequate human studies
Acetylsalicylic acid (low dose)	B	Yes	Yes (no adverse effects reported)	No teratogenic effects (inadequate human studies regarding the use of doses between 100–500 mg/day)
Cangrelor	C	Unknown	Unknown	No human data
Clopidogrel	B	Unknown	Yes	No adequate human data
Prasugrel	–	Unknown	Yes	Inadequate human data
Ticagrelor	–	Unknown	Yes	Inadequate human data; not recommended
Ticlopidine	C	Unknown	Yes	Inadequate human data
Vorapaxar	–	Unknown	Yes	Inadequate human data
Anticoagulants				
Acenocoumarol	D	Yes	Yes (no adverse effects reported)	Embryopathy (mainly first trimester),
Apixaban	–	Yes	Yes	No human data: not recommended
Dabigatran	–	Yes	Unknown	No human data; not recommended
Edoxaban	–	Unknown	Yes (contraindicated in breastfeeding)	Contraindicated; Hokusai-VTE study: 10 cases with exposure in first trimester, for up to 6 weeks. Results: six live births (four full term and two pre-term), one first trimester spontaneous abortion, and three elective terminations
Fondaparinux	–	Yes	Yes	Inadequate human data
Heparin (low molecular weight)	B	No	No	Retrospective cohort study with 693 live births: no increased risk of major developmental abnormalities
Heparin (unfractionated)	B	No	No	Human data: no fetus abnormalities
Phenprocoumon	D	Yes	Yes	Coumarin embryopathy
Rivaroxaban	–	Yes	Yes	Inadequate human data (contraindicated)
Warfarin	D	Yes	Yes	Coumarin embryopathy

In conclusion, intravascular imaging plays a key role in the diagnosis and management of ACS in women, allowing to differentiate SCAD from other causes of ACS and, in case of SCAD, to characterize its specific endotype and to guide medical therapy.

## Gender differences in participation in clinical trials: The who, what, why, when, how, and where

Women are still underrepresented in both early and later phase studies. The reasons for this phenomenon may be several. Traditionally, females were considered to have a more biological variability than males due to hormonal variations associated with estrous and menstrual cycles (131). Preclinical and clinical studies, recently, have refuted this theory, showing that females data are not more variable than those of males (132). Another reason could be that women affected by IHD are on average older than male counterparts and the enrollment of elderly patients in clinical trials has been historically low due to their frailty and comorbidities (133). Furthermore, women are less frequently referred to interventional treatment for ACS due to underestimation or misinterpretation of symptoms and are less likely to be treated with guideline-directed medical therapy (134). Interestingly, an RCT, enrolling 783 participants across 13 clinical centers, demonstrated that women present lower distrust of medical researchers and perceived greater risk of harm from trial participation than men (135). However, after disclosure of investigator patent ownership or monetary incentives, willingness to participate increased more in women than in men. This suggests that female aversion for participating in a scientific experiment could be, at least partially, overcomed by active and informed involvement in trial’s participation. Lastly, the research for sex differences may not necessarily involve a doubling of the pre-determined sample size (and costs) in order to reach an adequate statistical power. A recent statistical model, using factorial designs and tested for now only in animal studies, revealed necessary increases of only 14–33% to include both sexes, even after statistical correction for the use of multiple factors (136). Nevertheless, further studies are needed to validate this model in clinical trials.

## Conclusion

Thirty one year after defining the “Yentl syndrome,” women are still understudied, underdiagnosed and undertreated. Their representation in RCTs is still too low (at most 30% of the overall trial population), although their pharmacodynamic and pharmacokinetic responses to antithrombotic drugs and their baseline bleeding and ischemic risks may differ significantly from males. Furthermore, investigations about antiplatelet drug safety and efficacy should not end with regulatory approval. Phase 4 studies, real-world data and systematic adverse-event reporting are critical to detect bleeding, ischemic events and off-target toxicities. A recent report from Hilleary et al. compared the proportion of females with an established diagnosis of IHD that received patient education, in terms of diet, exercise, tobacco use and weight reduction, with the corresponding proportion of males (137). Surprisingly, it revealed that a lower proportion of women received patient education related to managing cardiovascular risk, after adjusting for covariates. Accordingly, a lower proportion of women reaches cardiovascular risk factor target levels, as EUROASPIRE V registry has recently showed (138). Overall, a gender gap still exists for risk factor target management in secondary prevention, mostly in disfavor of women. Therefore, more deliberate and intentional effort needs to be performed in closing this gender gap, especially since risk factors like smoking and diabetes may have an even more detrimental effect in female patients, as mentioned above. In the era of precision medicine, it is unacceptable that women are treated “just like men” and viewed as a negligible minority. Historically, women’s health research has focused on reproductive health, a phenomenon known as “bikini medicine” (139). Now, it’s time that cardiovascular research efforts move away from mere awareness about gender differences to palpable and concrete action. “Go Red for Women” campaign, launched in 2004 by the American Heart Association, is pushing in this direction, in order to increase awareness and foster specific guidelines for prevention and treatment of IHD in women. Recently, POPular AGE trial and ELDERLY-ACS trial have evaluated safety and efficacy of different anti-platelet regimens in elderly, a clinical minority under-represented in RCTs, as women (140–142). Similarly, RCTs recruiting a significant proportion of women could be the solution to overcome the “Yentl syndrome.”

## Author contributions

All authors listed have made a substantial, direct, and intellectual contribution to the work, and approved it for publication.
